# Factors associated with positive blood cultures in children in nine African and Asian countries: the ACORN2 surveillance network

**DOI:** 10.1136/bmjgh-2025-020448

**Published:** 2025-10-20

**Authors:** Cristina Ardura-Garcia, Jill Hopkins, Sue J Lee, Naomi Waithira, Chris Painter, Clare L Ling, Tamalee Roberts, Thyl Miliya, Noah Obeng-Nkrumah, Japheth A Opintan, Emmanuel P Abbeyquaye, Raph L. Hamers, Yulia R Saharman, Robert Sinto, Mulya R Karyanti, R Fera Ibrahim, Samuel O Akech, Anousone Duangnouvong, Khamla Choumlivong, Nicholas A Feasey, Diana Kululanga, Samantha Lissauer, Abhilasha Karkey, Narayan Kunwar, Justice E Erakhaiwu, Iruka N Okeke, Ini Adebiyi, Abiodun B Oduola, Babatunde O Ogunbosi, Olukemi O Tongo, Ifeoma A Ude, Oladipo Aboderin, Adeyemi T Adeyemo, Sylvester S Edward, Ugowe Osagie, Hoa Nguyen Thi, Pham Ngoc Thach, Tran Van Giang, Lan Huong Hoang Thi, Huu Tung Trinh, H. Rogier van Doorn, Elizabeth A Ashley, Paul Turner

**Affiliations:** 1 Cambodia Oxford Medical Research Unit, Angkor Hospital for Children, Siem Reap, Cambodia; 2 Centre for Tropical Medicine and Global Health, University of Oxford Nuffield Department of Medicine, Oxford, UK; 3 Mahidol-Oxford Tropical Medicine Research Unit, Mahidol University Faculty of Tropical Medicine, Bangkok, Thailand; 4 Department of Infectious Diseases, The Alfred Hospital and School of Translational Medicine, Monash University, Melbourne, Victoria, Australia; 5 Lao-Oxford-Mahosot Hospital-Wellcome Trust Research Unit, Vientiane, เวียงจันทน์, Lao People’s Democratic Republic; 6 Department of Microbiology, University of Ghana School of Biomedical and Allied Health Sciences, Accra, Ghana; 7 Department of Medical Microbiology, University of Ghana Medical School, Accra, Ghana; 8 Paediatric Division, 37 Military Hospital, Accra, Ghana; 9 Oxford University Clinical Research Unit Indonesia, Faculty of Medicine, Universitas Indonesia, Jakarta Pusat, Jawa Barat, Indonesia; 10 Department of Microbiology, Faculty of Medicine Universitas Indonesia, Dr. Cipto Mangunkusumo National Hospital, Jakarta, Indonesia; 11 PT PELNI Hospital, Jakarta, Jakarta, Indonesia; 12 Division of Tropical and Infectious Diseases, Department of Internal Medicine, Faculty of Medicine Universitas Indonesia, Dr. Cipto Mangunkusumo National Hospital, Jakarta, Indonesia; 13 Department of Child Health, Faculty of Medicine Universitas Indonesia, Dr. Cipto Mangunkusumo National Hospital, Jakarta, Indonesia; 14 University of Indonesia Hospital, Jakarta, Indonesia; 15 KEMRI-Wellcome Trust Research Programme, Kilifi, Kenya; 16 Microbiology Laboratory, Mahosot Hospital, Lao-Oxford-Mahosot Hospital-Wellcome Trust Research Unit, Vientiane, Lao People’s Democratic Republic; 17 Setthathirath Hospital, Vientiane, Lao People’s Democratic Republic; 18 Malawi Liverpool Wellcome Programme, Kamuzu University of Health Sciences, Blantyre, Malawi; 19 University of St Andrews School of Medicine, St Andrews, UK; 20 Department of Clinical Sciences, Liverpool School of Tropical Medicine, Liverpool, UK; 21 University of Liverpool Institute of Infection and Global Health, Liverpool, UK; 22 Oxford University Clinical Research Unit Nepal, Lalitpur, Nepal; 23 Department of Medical Microbiology and Parasitology, University of Ibadan College of Medicine, Ibadan, Nigeria; 24 Department of Pharmaceutical Microbiology, Faculty of Pharmacy / Department of Medical Microbiology and Parasitology, University of Ibadan College of Medicine, Ibadan, Nigeria; 25 Department of Medical Microbiology and Parasitology, University College Hospital Ibadan, Ibadan, Nigeria; 26 University College Hospital Ibadan, Ibadan, Nigeria; 27 University of Ibadan, Ibadan, Nigeria; 28 Department of Padiatrics, University of Ibadan College of Medicine, Ibadan, Nigeria; 29 Paediatric Infectious Diseases Unit, University College Hospital Ibadan, Ibadan, Nigeria; 30 Obafemi Awolowo University College of Health Sciences, Ile-Ife, Nigeria; 31 Obafemi Awolowo University Teaching Hospital Complex, Ile-Ife, Nigeria; 32 Oxford University Clinical Research Unit, Hanoi, Viet Nam; 33 National Hospital of Tropical Diseases, Hanoi, Viet Nam; 34 Hanoi Medical University, Hanoi, Viet Nam; 35 Hue Central Hospital, Hue, Viet Nam; 36 Children’s Hospital 2, Ho Chi Minh City, Viet Nam

**Keywords:** Global Health, Africa South of the Sahara, Blood disorders, Other diagnostic or tool, Paediatrics

## Abstract

**Background:**

Blood culture (BC) in children has relatively low diagnostic yield and high contamination rates, limiting cost-effectiveness. We aimed to determine readily available baseline characteristics to identify hospitalised children with a likelihood of higher diagnostic yield in low- and middle-income countries.

**Methods:**

We used data from ACORN2, a prospective clinical surveillance network including 19 hospitals across Africa and Asia. We included participants <18 years, hospitalised for a suspected infection, prescribed parenteral antibiotics and with a BC sample. Sociodemographic and clinical data were recorded for each infection episode and linked to routine microbiology data. We described true pathogen (non-contaminant) BC positivity proportion and performed mixed-effects logistic regression, with study site and patient as the random effect, to identify factors associated with BC positivity.

**Results:**

Of the 26 407 paediatric infection episodes, 17 815 (67%) had a BC sample and 15 384 were included in the analysis. BC results were: true pathogens in 689 (4.5%), contaminants in 1399 (9%) and uncertain pathogens in 143 (0.9%). In the multivariable model, factors associated with a positive BC were age (29 days–12-month-olds OR 1.33, 95% CI 1.06 to 1.66 and 5–18 year-olds OR 1.62, 95% CI 1.30 to 2.01 vs 1–4 year-olds), number of clinical severity signs (OR 1.29, 95% CI 1.18 to 1.40 per one sign) and hospital acquired infection (OR 3.05, 95% CI 2.30 to 4.06 vs community-acquired). Suspected diagnosis of sepsis (OR 2.09, 95% CI 1.67 to 2.61), gastrointestinal/abdominal (OR 2.36, 95% CI 1.78 to 3.13), skin and soft tissue or bone (OR 3.64, 95% CI 2.57 to 5.14) and genitourinary infection (OR 2.22, 95% CI 1.39 to 3.56) were more likely to have a positive BC, compared with respiratory infections.

**Conclusion:**

We confirmed the low BC yield among hospitalised children. We identified groups for which diagnostic stewardship efforts to increase BC uptake should be prioritised and others in which it could be limited in times of financial or logistic constraints.

**WHAT IS ALREADY KNOWN ON THIS TOPIC**

**WHAT THIS STUDY ADDS**

- This study confirmed a low blood culture yield in hospitalised children in LMICs (4.5%) with a 2-fold higher contamination rate (9%).
- We showed that non-laboratory factors such as age (1–12 month-olds and 5–18 year-olds), clinical severity signs (temperature, heart rate, mental status and peripheral perfusion), suspected diagnosis other than respiratory infection and having a hospital-acquired infection may identify children with a higher proportion of blood culture positivity.

**HOW THIS STUDY MIGHT AFFECT RESEARCH, PRACTICE OR POLICY**

- This study highlights the potential opportunity to optimise blood culture sampling in children.
- The factors identified in this study are routinely collected in most settings and may be used by policy makers and healthcare workers to develop protocols to optimise BC indications in hospitalised children in LMICs.

## Introduction

Blood culture (BC) has a low positive yield overall and is even lower in neonates and children.^1^,^8^ However, BC is the gold standard diagnostic method for bloodstream infections (BSIs), which are associated with high mortality in neonates and children.^9^,^11^ Published data on BC yield in children with suspected infection vary greatly from <1% to over 50%,^1^,^8^ depending on the setting, age, year performed (post *Haemophilus influenzae* type B and pneumococcal conjugate vaccine era) and inclusion criteria (hospitalised, sicker children). BCs are recommended in clinical practice guidelines for severe focal infections, such as pneumonia, skin and soft tissue (SST) or bone infections,^12^,^14^ to attempt to identify the causative bacteria and guide targeted antibiotic treatment. As a result, they are performed in up to half of children hospitalised for suspected infections, depending on the setting and diagnosis.^4 15 16^ In addition, due to sampling difficulties, the contamination rate (positive BC with a non-pathogenic microbe) in paediatric BCs is often up to twofold to fourfold higher than the true-positive rate.^7 17 18^ Performing BCs that are not informative for patient management results in increased costs, longer hospital stays and suboptimal antibiotic use.^18^,^20^

Targeting BCs to cases with a higher positive yield may enhance their cost-effectiveness and availability, particularly in resource-limited settings. The recent BC bottle shortage in the USA led to recommendations to reduce BC testing in low yield cases.^21^ Laboratory supply shortages, limited availability and the need for patients to pay out of pocket are recurrent issues in low- and middle-income country (LMIC) settings, which together with the high cost of an individual BC, highlight the need for more cost-effective BC testing. There are limited data on factors associated with BC positivity in children in LMICs. Existing severity prediction scores may identify children at risk of poor outcomes,^22^,^24^ but do not identify children with bacteraemia,^25 26^ and they include measurements that are not always available in LMICs. Most paediatric studies on predictors of BC positivity have focused on specific bacterial species,^27^ infection syndromes,^28^ comorbidities (eg, febrile neutropenia)^29^ or age groups^30^ or were performed before the extensive implementation of pneumococcal conjugate vaccine.^7^ Laboratory tests, such as white blood cell count with differential or C-reactive protein, have also been associated with paediatric bacteraemia,^8 28 31 32^ but these are not always available in LMICs and the turnaround time may be too long for initial decision-making on need for BC testing. Clinical characteristics, such as lethargy, prolonged fever, comorbidities and hospitalisation, may also predict BC positivity in children,^3 5^ though evidence is scarce. In addition, specific focal infections such as SST or respiratory infections^2833^,^37^ have a lower BC yield, while infants and neonates have a higher yield than older children.^38 39^ It may therefore be possible to determine readily available factors that may help optimise BC sampling. We aimed to identify clinical factors associated with BC positivity in hospitalised children, using data from a multicentre antimicrobial resistance surveillance study from LMICs.

## Methods

### Study design and setting

A Clinically Oriented Antimicrobial Resistance Surveillance Network 2 (ACORN2) is an international prospective clinical surveillance study incorporated into the routine workflow in 19 hospitals from nine LMICs (Cambodia, Ghana, Indonesia, Kenya, Laos, Malawi, Nepal, Nigeria and Vietnam).^11 40^ In brief, participating hospitals collected sociodemographic data, clinical features, microbiology results and management data and outcomes of hospitalised patients treated with parenteral antibiotics for clinically suspected acute infection (online supplemental figure 1). The study was approved by the Oxford Tropical Research Ethics Committee (OxTREC 524–21), with additional approvals from local or national ethics committees for each hospital as required.

### Study population

For this analysis, we included children under 18 years old enrolled into ACORN2 who had a valid blood sample sent for culture. We included both community-acquired infections (CAIs) and hospital-acquired infections (HAIs). We excluded children who had been transferred in from other health centres, those with an HAI where the date of symptom onset was less than 2 days after date of admission and those with a presence of malaria parasites.

### Study procedures

Selected wards were screened daily to identify eligible participants with CAI and by weekly point prevalence surveys to identify HAI. Sociodemographic and clinical data were entered into a REDCap database.^41 42^ Prior to study commencement, site diagnostic microbiology laboratories were audited to determine readiness for surveillance, with corrective actions implemented to ensure alignment with accepted international quality standards. At the time of assessment, using the ACORN2 laboratory assessment tool, 12 laboratories were using a BD Bactec, four a BioMerieux BacT/Alert, two had both Bactec and Bact/Alert systems and two were using manual methods.^11^ Clinical and nursing staff were trained in the appropriate collection and transport of diagnostic microbiology specimens before the start of the study, with real-time monitoring via the project dashboard and quarterly follow-up of specimen processing at laboratory quality management meetings. This information and more can be found in the study′s diagnostic stewardship manual (https://doi.org/10.6084/m9.figshare.27900042.v2). Routine microbiology data were exported from laboratory information systems or WHONET files from each centre. Microbiology data were standardised and merged with clinical data via a RShiny app.^43^ Patients and infection episodes for each patient were assigned a unique identifier. Specimens were identified using the patient identifier and a specimen collection date and were linked to an infection episode if collected within two calendar days of the admission for CAI or if collected on or up to two calendar days following the day of symptom onset for HAI.

### Definitions of exposures

We selected factors potentially associated with BC positivity based on previous literature^3 5 7 8 25 26 28 30^ and information available in the ACORN2 database^44^ . The selected factors included sociodemographics, recent healthcare exposure (previous 3 months), comorbidities and admission characteristics from the admission form, as well as suspected diagnosis, CAI versus HAI, clinical severity, BC timing (first 24 hours or later) and antibiotic exposure (24 hours before BC yes/no) from the infection episode form. Sociodemographic information included country, age and sex. Age was classified into four groups: neonates (<29 days), infants (29 days to 12 months), 1–4 year-olds and 5–18 year-olds, following frequently used age groups in paediatrics and the age distribution of the study sample. We included HIV/AIDS, tuberculosis, malnutrition, diabetes, cancer and heart, respiratory, neurologic, gastrointestinal (GI), hepatic, renal and rheumatologic or connective tissue diseases as ‘any comorbidity yes/no’. Suspected diagnoses were classified into respiratory infection, sepsis, GI or abdominal, central nervous system (CNS), SST or bone, genitourinary infection and other or unknown. Sepsis was defined as clinical sepsis with an unclear source, without an obvious source or when the source was not specified. The complete definition of clinical sepsis included in the study′s diagnostic stewardship manual may be found in the Supplementary Materials. HAIs were defined as a clinically suspected infection with symptom onset occurring >48 hours after admission and with prescription of new parenteral antibiotics; the rest were classified as CAI (including recent healthcare exposure). Clinical signs used for severity classification were abnormal temperature, tachycardia, altered mental status and reduced peripheral perfusion for every child (‘sepsis six’ for children,^45^ together with reduced activity, feeding difficulties and convulsions for neonates (WHO general danger signs for neonates)).^46^ We used each sign separately and as a continuous clinical severity score of 0–4 points (plus 0–7 points for neonates). For HAI, we recorded admission to the intensive care unit >48 hours, surgery during admission and medical devices as potential factors associated with BC positivity. Medical devices included peripheral venous catheter (PVC), central nervous catheter (CVC), urinary catheter and intubation, both independently and as a composite exposure variable (any medical device yes/no).

### Definitions of outcomes

The outcome of interest for this analysis was a positive BC from an independent infection episode. BC results were classified into negative, contaminant, uncertain and pathogen. Negative BCs included reported results of no (significant) growth, no organism name, no culture result or blank. Contaminant bacteria included *Bacillus* spp (not *anthracis*), coagulase-negative staphylococci (lack of prematurity and medical devices), *Corynebacterium* spp (not *diphtheriae*), diphtheroids, *Micrococcus* spp, *Cutibacterium* and unidentified bacteria with only Gram stain result. Pathogens included WHO-GLASS / antimicrobial resistant priority organisms,^47^ miscellaneous Enterobacterales (defined as human pathogens in UK Standards for Microbiology Investigations ‘Identification of Enterobacteriaceae’),^48^ WHO Global Invasive Bacterial Vaccine-Preventable Disease/meningitis organisms,^49^ HACEK group, *Brucella, Vibrio, Yersinia, Franciscella*, beta-haemolytic *Streptococci* and *Enterococci*, *Burkholderia pseudomallei, Chromobacterium*, *Candida* (or ‘yeast’) and *Cryptococcus*. All other growth was defined as uncertain significance (online supplemental table 1).

### Statistical analyses

Demographic and clinical data were tabulated and summarised using descriptive statistics, by BC positivity. We performed a mixed-effects logistic regression analysis to identify predictors of BC positivity (vs negative) per infection episode. We included study site and patient id as random effects to allow for different intercepts. All the variables described under ‘exposures’ were considered. A positive BC was defined as the presence of a ‘pathogen’, and the rest were defined as negative. When there was more than one identified bacterial species per infection episode per child, the infection episode’s BC was classified as positive if any were identified as ‘pathogen’, and negative in all other cases. Infection episodes with more than one isolated species classified as ‘pathogen’ were included as positive BCs (n=16). We constructed two multivariable models: one ‘simple’ model including exposures associated with BC positivity in univariable analysis with a p value ≤0.1 and one ‘stepwise’ model using automated stepwise backward selection methods. The final model was selected based on Akaike’s information criterion (AIC) and the log likelihood. Missing values were excluded from the analyses. Information available per variable is shown in table 1. We report (adjusted) ORs ((A)ORs) with 95% CIs. R statistical programme V.4.3.0 was used for computation.^50^

**Table 1:** Characteristics of included children per infection episode

Characteristic	N	All (n=15 384)	Blood culture results (row percentages, n (%))		P value
			Negative (n=14 695, 95.5%)	Positive (n=689, 4.5%)	
Sociodemographics					
Region	15 384				<0.001
Africa		8391 (55)	7917 (94.4)	474 (5.6)	
Asia		6993 (45)	6778 (96.9)	215 (3.1)	
Country	15 384				<0.001
Ghana		731 (4.8)	688 (94.1)	43 (5.9)	
Indonesia		395 (2.6)	355 (89.9)	40 (10.1)	
Kenya		3912 (25)	3808 (97.3)	104 (2.7)	
Cambodia		3152 (20)	3061 (97.1)	91 (2.9)	
Laos		1927 (13)	1875 (97.3)	52 (2.7)	
Malawi		2391 (16)	2236 (93.5)	155 (6.5)	
Nigeria		1357 (8.8)	1185 (87.3)	172 (12.7)	
Nepal		1282 (8.3)	1257 (98.0)	25 (2.0)	
Vietnam		237 (1.5)	230 (97.0)	7 (3.0)	
Age (years), median (IQR)	15 384	1.1 (0.1–4.1)	1.1 (0.1–4.0)	1.0 (0.1–5.9)	0.900
Age groups	15 384				<0.001
<29 days		3318 (22)	3141 (94.7)	177 (5.3)	
29 days-12 months		3425 (22)	3272 (95.5)	153 (4.5)	
1–4 years		5173 (34)	5008 (96.8)	165 (3.2)	
5–18 years		3468 (23)	3274 (94.4)	194 (5.6)	
Female sex	15 372	6656 (43)	6358 (95.5)	298 (4.5)	>0.9
Recent HC exposure	15 259	3046 (20)	2917 (95.8)	129 (4.2)	0.500
Comorbidities	15 384				
Any		2744 (18)	2604 (94.9)	140 (5.1)	0.082
Cancer		421 (2.7)	397 (94.3)	24 (5.7)	0.200
Malnutrition		442 (2.9)	414 (93.7)	28 (6.3)	0.056
Infection episode					
Hospital acquired infection	15 384	435 (2.8)	358 (82.3)	77 (17.7)	
Suspected diagnosis	15 384				<0.001
Respiratory infection		5396 (35)	5260 (97.5)	136 (2.5)	
Sepsis		4516 (29)	4214 (93.3)	302 (6.7)	
GI or abdominal infection		1308 (8.5)	1226 (93.7)	82 (6.3)	
CNS infection		979 (6.4)	952 (97.2)	27 (2.8)	
SST or bone infection		585 (3.8)	533 (91.1)	52 (8.9)	
Genitourinary infection		365 (2.4)	343 (94.0)	22 (6.0)	
Other or unknown		2235 (15)	2167 (97.0)	68 (3.0)	
Clinical severity characteristics					
No clinical severity characteristics	15 384	5993	5786 (96.5)	207 (3.5)	<0.001
Abnormal temperature	15 187	5421 (36)	5118 (94.4)	303 (5.6)	<0.001
Tachycardia	14 784	3456 (23)	3274 (94.7)	182 (5.3)	0.004
Altered mental status	15 013	3444 (23)	3267 (94.9)	177 (5.1)	0.021
Reduced peripheral perfusion	14 268	1737 (12)	1652 (95.1)	85 (4.9)	0.4
Reduced activity (neonates)	3165	596 (19)	546 (91.6)	50 (8.4)	<0.001
Feeding difficulty (neonates)	3084	918 (30)	839 (91.4)	79 (8.6)	<0.001
Convulsions (neonates)	3188	112 (3.5)	98 (87.5)	14 (12.5)	<0.001
Clinical severity score, median (IQR)	13 455	1 (0–2)	1 (0–2)	1 (0–2)	<0.001
BC within 24 hours of admission	15 318	14 677 (96)	14 017 (95.5)	660 (4.5)	0.6
Antibiotic 24 hours before BC	13 559	3527 (26)	3397 (96.3)	130 (3.7)	0.002

BC, blood culture; CI, Confidence Interval; CNS, central nervous system; GI, gastrointestinal; HC, health care; IQR, interquartile range; SST, skin and soft tissue infection.

We described the pathogens found in infection episodes where none of the risk factors for BC positivity were present. We performed a sensitivity analysis, in which BCs classified as ‘uncertain’ were also defined as a positive BC. We also performed two subgroup analyses: one including only neonates (specified a priori) and another including only respiratory infections. The model for respiratory infections included only study site as random effect due to lack of convergence. Factors associated with BC positivity for HAI were reported in a descriptive table, but no further regression analysis was performed, as HAIs were not the main focus of this study and the number of positive BCs among this group was small, limiting any multivariable analysis.

### Patient and public involvement

There was no patient or public involvement in the design and conduct of this study.

## Results

**Figure 1:**
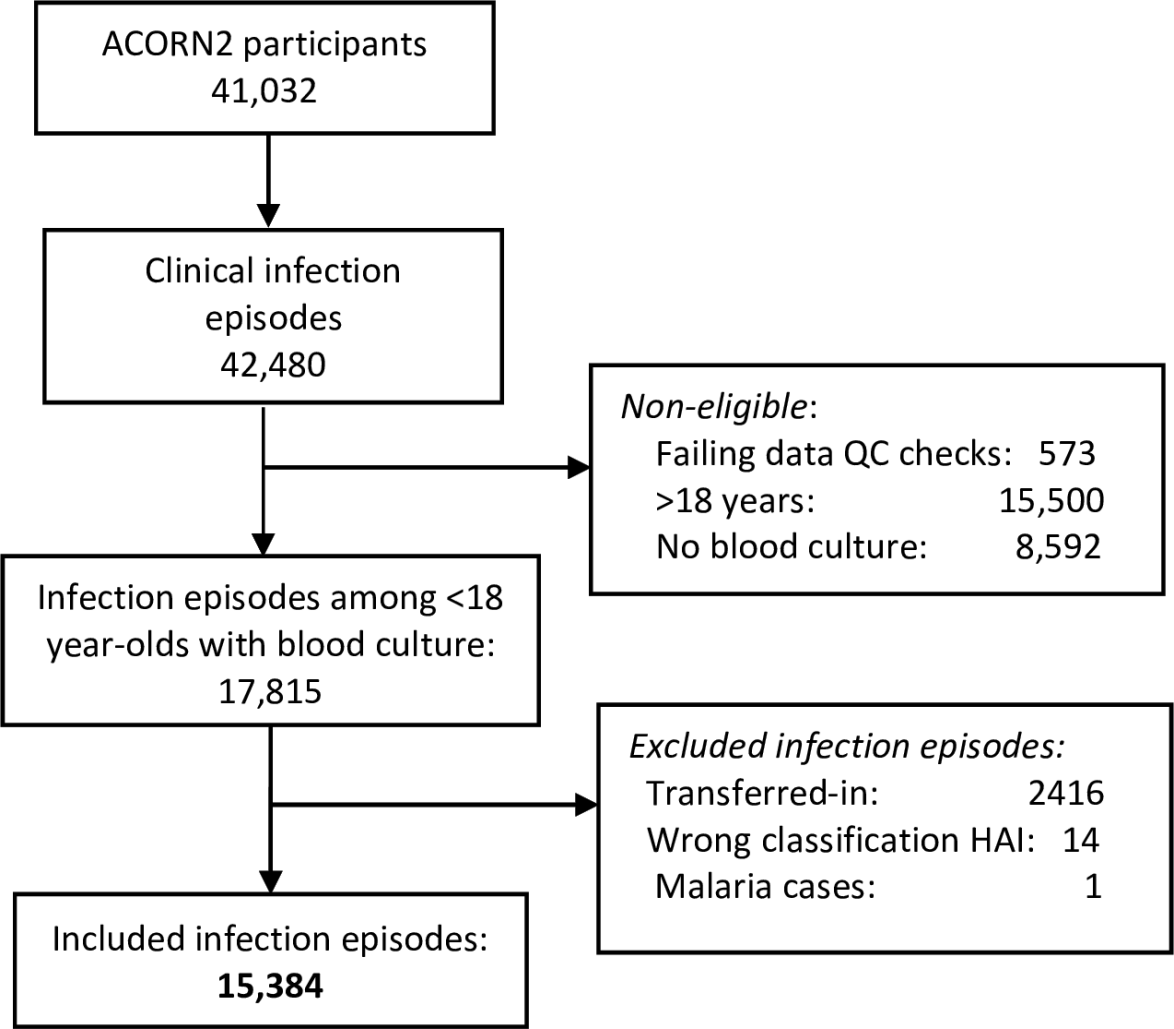
Study population. HAI, hospital acquired infection; QC, quality control.

Between 7 September 2021 and 31 March 2024, 41 032 patients were enrolled into ACORN2, including 26 407 paediatric infection episodes (from 26 000 paediatric patient admissions), of which 17 815 (67.5%) had a BC sample and 15 384 were included in this analysis (figure 1). Characteristics of the included infection episodes are summarised in table 1. Infection episodes were evenly distributed between African and Asian countries. Median age was 1.1 years (IQR: 0.1–4.1 years), including 3318 infections in neonates (21.6%), and 43.3% of the infections occurred in female children. Associated comorbidities were reported in 17.8% (2744) of infection episodes, the most common being malnutrition (442, 2.9%) and any type of cancer (421, 2.7%). One fifth (3,046) reported healthcare exposure during the previous 3 months and 435 (2.8%) were classified as HAI. The most common suspected diagnoses were respiratory infections (5396, 35.1%) and sepsis (4516, 29.4%). Among the clinical severity characteristics, the most common was an abnormal temperature (5421, 35.7%) and feeding difficulties among infection episodes in neonates (918, 29.8%). The median clinical severity score was 1 (IQR 0–2). Nearly all BCs were taken within the first 24 hours of admission or of symptom onset for HAI, and 26% (3527) of infections had received antibiotics in the 24 hours preceding the BC collection. Of the 435 HAI episodes, 74% (323) had a medical device in situ at HAI symptom onset, a PVC being the most common (301, 69.2%), with 5.3% having a CVC (23) and 7.4% a urinary catheter^32^ (online supplemental table 2).

**Figure 2:**
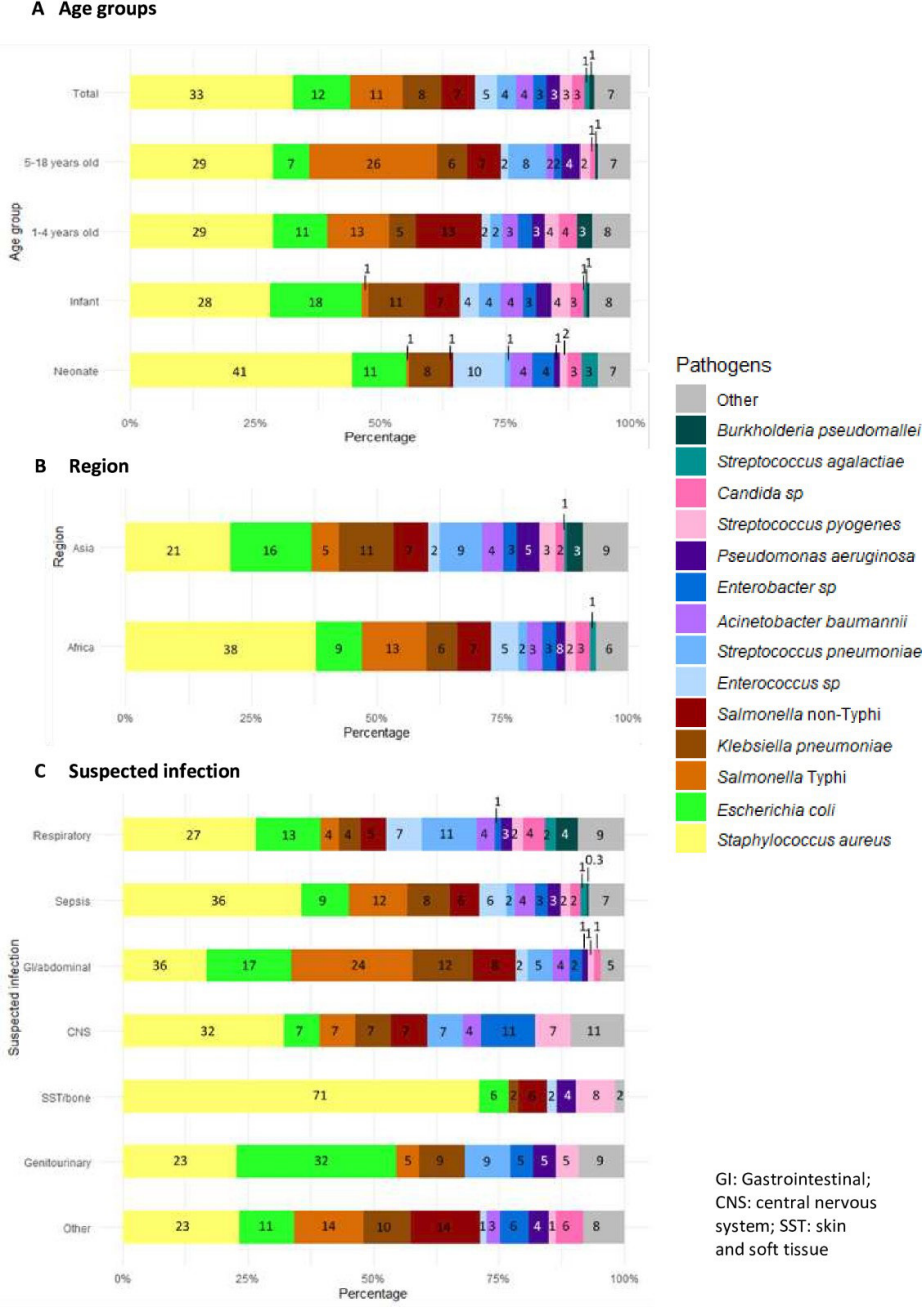
Pathogens identified from the 689 positive blood cultures, by age group (**A**), by region (**B**) and by suspected infection (**C**). (n=705).

Out of the 15 384 infection episodes with a BC sample, 689 (4.5%) were positive for a pathogen. In 16 infection episodes, two bacterial species were identified as pathogens (total n=705). Contamination was identified in 9.1% of BCs (1399) and an uncertain pathogen in 0.9% (143). The most common pathogen was *Staphylococcus aureus* (229/705, 32.5%), followed by gram-negative bacilli: *Escherichia coli* (81, 11.5%), *Salmonella* Typhi (74, 10.5%), *Klebsiella pneumoniae* (54, 7.7%) and non-Typhi *Salmonella* sp (47, 6.7%). Distribution of pathogens varied by age with *S. aureus* being identified in 41.3% of positive BCs in neonates, *E. coli* in 18.4% in infants and *S*. Typhi in 25.5% in 5–18 year-olds. Distribution of pathogens also varied by region, with a larger proportion of *S. aureus* in African countries (37.8% vs 20.8% in Asian countries) and of Enterobacterales in Asian countries (*E. coli:* 16.3% vs 9.3% in African countries and *K. pneumoniae*: 10.9% vs 6.2% in Africa). *Burkholderia pseudomallei* was exclusively found in Asian countries (7, 3.2% of positive BCs) (figure 2 & online supplemental table 1).

**Figure 3:**
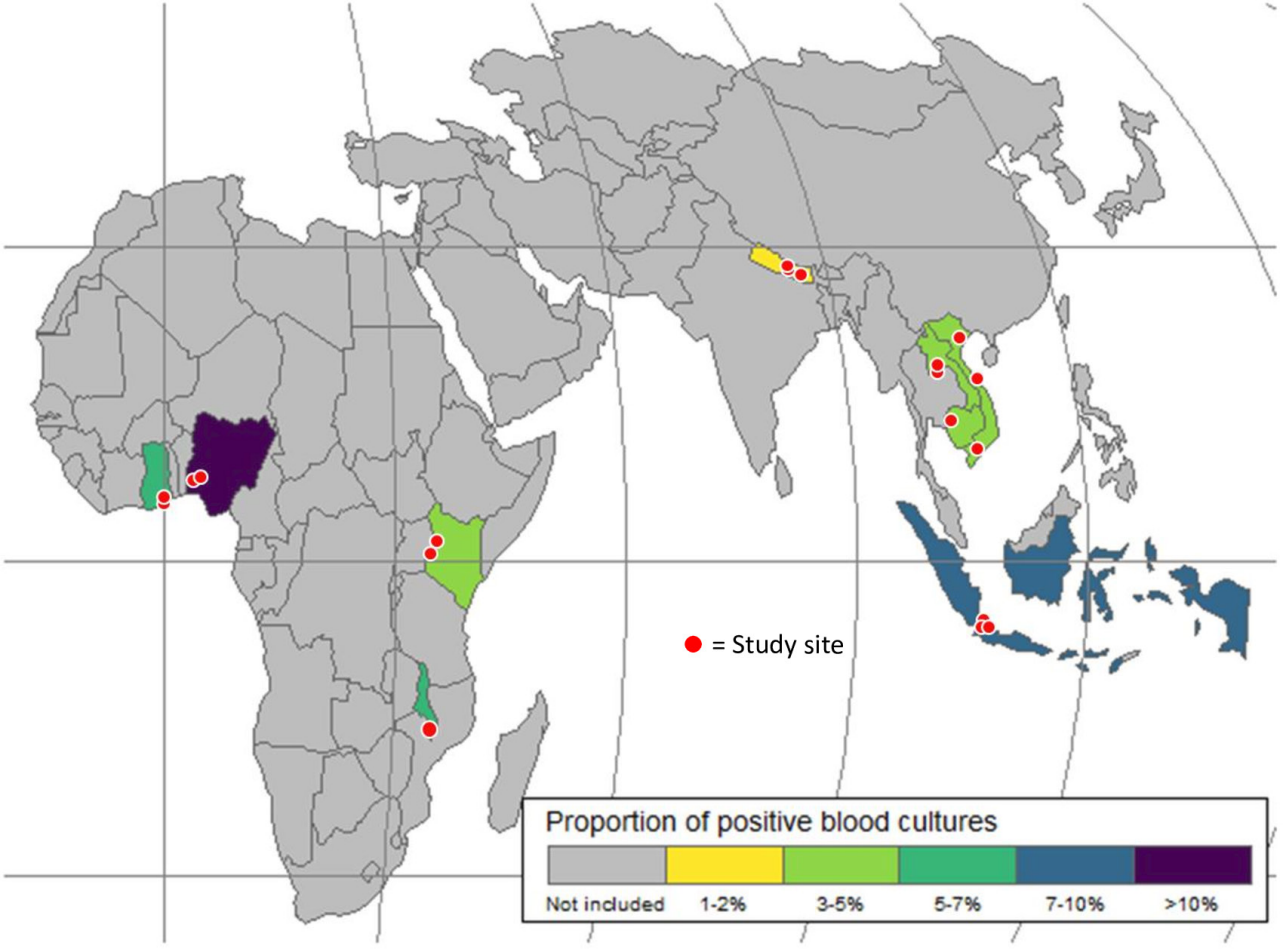
Countries and sites participating in ACORN2 and proportion of blood culture positivity.

The proportion of positive (true pathogen) BCs varied by location from 2.0% in Nepal to 13% in Nigeria, averaging 3.1% (215/6993) in Asian countries and 5.6% (474/8391) in African countries (figure 3B, table 1). The distribution of BC positivity rate also varied by age, being higher in neonates (177, 5.3%) and infants (153, 4.5%), with a decrease among 1–4 year-olds (165, 3.2%), to increase again among 5–18 year-olds (194, 5.6%), with no differences by sex. BC yield was higher among children with HAI (17.7% vs 4.1%, 612/14949 among CAI). BC positivity rates also differed by initial diagnosis, with respiratory infections presenting the lowest (2.5%) and SST or bone infections being the highest (8.9%).

In the univariate mixed-effects model, age group, HAI, diagnosis of suspected infections other than respiratory infections and each of the clinical severity characteristics were associated with BC positivity (table 2). All four clinical severity characteristics and the continuous composite clinical severity score were associated with BC positivity (table 2). We included only the composite clinical score in the multivariable model, as it had the lowest AIC when compared with models that included each severity characteristic separately. The best performing multivariable model (simple model) showed that 29 days–12-month-olds (AOR 1.33, 95% CI 1.06 to 1.66) and 5–18 year-olds (AOR 1.62, 95% CI 1.30 to 2.01) compared with 1–4 year-olds, and children with signs of clinical severity (AOR 1.29, 95% CI 1.18 to 1.40 per each additional sign) had higher odds of a positive BC. The odds of positive BC were three times higher for HAI when compared with CAIs (AOR 3.05, 95% CI 2.30 to 4.06). From the suspected diagnoses, sepsis (AOR 2.09, 95% CI 1.67 to 2.61), GI or abdominal (AOR 2.36, 95% CI 1.78 to 3.13), SST or bone (AOR 3.64, 95% CI 2.57 to 5.14) and genitourinary infection (AOR 2.22, 95% CI 1.39 to 3.56) were associated with BC positivity when compared with respiratory infections.

**Table 2:** Mixed effects logistic regression analysis for factors associated with blood culture positivity for all children under 18 years old participating in ACORN study (study site and patient id as random effect)

Characteristic	Univariate			Multivariable		
	OR	95% CI	P value	AOR	95% CI	P value
Sociodemographics						
Age groups						
<29 days	1.13	0.90 to 1.42	0.275	0.97	0.76 to 1.23	0.812
29 days–12 months	**1.37**	**1.09 to 1.71**	**0.006**	**1.33**	**1.06 to 1.66**	**0.014**
1–4 years	—	—		—	—	
5–18 years	**1.75**	**1.42 to 2.17**	**<0.001**	**1.62**	**1.30 to 2.01**	**<0.001**
Female sex	0.96	0.82 to 1.12	0.607			
Recent HC exposure	1.04	0.82 to 1.31	0.754			
Comorbidities						
Any	1.14	0.92 to 1.41	0.231			
Cancer	1.36	0.86 to 2.14	0.188			
Malnutrition	1.28	0.84 to 1.95	0.246			
Infection episode						
Hospital acquired infection	**3.18**	**2.40 to 4.21**	**<0.001**	**3.05**	**2.30 to 4.06**	**<0.001**
Suspected diagnosis						
Respiratory infection	—	—		—	—	
Sepsis	**2.13**	**1.71 to 2.65**	**<0.001**	**2.09**	**1.67 to 2.61**	**<0.001**
GI or abdominal infection	**2.38**	**1.80 to 3.16**	**<0.001**	**2.36**	**1.78 to 3.13**	**<0.001**
CNS infection	0.82	0.54 to 1.24	0.340	0.74	0.49 to 1.13	0.166
SST or bone infection	**3.71**	**2.64 to 5.21**	**<0.001**	**3.64**	**2.57 to 5.14**	**<0.001**
Genitourinary infection	**2.43**	**1.52 to 3.88**	**<0.001**	**2.22**	**1.39 to 3.56**	**<0.001**
Other or unknown	**1.40**	**1.03 to 1.91**	**0.032**	1.26	0.93 to 1.72	0.136
Clinical characteristics						
Abnormal temperature	**1.56**	**1.33 to 1.84**	**<0.001**			
Tachycardia	**1.27**	**1.06 to 1.52**	**0.009**			
Altered mental status	**1.32**	**1.07 to 1.62**	**0.009**			
Reduced peripheral perfusion	**1.47**	**1.14 to 1.90**	**0.003**			
Number of clinical severity signs*	**1.28**	**1.18 to 1.39**	**<0.001**	**1.29**	**1.18 to 1.40**	**<0.001**
BC within 24 hours of admission	1.36	0.91 to 2.03	0.136			
Antibiotic 24 hours before BC	1.20	0.91 to 1.57	0.191			

Values in bold show associations with p value <0.05 or CI which does not cross 1.

* Clinical severity criteria for neonates were not included in the analysis of the whole cohort, but analysed only for the neonatal subgroup analysis (online supplemental table 5).

AOR, adjusted odds ratio; BC, blood culture; CI, Confidence Interval; CNS, central nervous system; GI, gastrointestinal; HC, health care; OR, Odds Ratio; SST, skin and soft tissue infection.

Next, we described the pathogens identified among children with none of the identified risk factors associated with BC positivity (see above, (online supplemental table 3). Among 1–4 year-olds with suspected community-acquired respiratory infection, there were 9/743 (1.2%) positive BCs if no clinical severity characteristic, and 18/818 (1.0%) if ≤1 clinical severity characteristic. The specific pathogens found in the latter group included only two cases of *S. pneumoniae*, as well as five *S. aureus*, two each of *B. pseudomallei*, yeast and *Salmonella* non-Typhi and one each of *E. faecium, E. coli*, Gr. A streptococcus, *Mycobacterium* and *Salmonella* Typhi.

The sensitivity analysis redefining BCs with ‘uncertain pathogen’ as positive (n=832 positive BCs) returned the same associated factors as the main analysis, though with weaker associations (online supplemental table 4). For the neonates’ subgroup analysis, we still found a strong association between HAI (AOR 4.46, 95% CI 2.56 to 7.77) and BC positivity (online supplemental table 5). Clinical severity signs were also independently associated with an increased odds of BC positivity of 1.18 (95% CI 1.05 to 1.32) for each added sign from the seven included in the clinical neonatal severity score. Sepsis (AOR 1.68, 95% CI 1.01 to 2.81) and SST or bone infection (AOR 2.64, 95% CI 1.01 to 6.88) were also associated with increased odds of BC positivity compared with respiratory infections. To better characterise the role of BC samples for respiratory infections in children, we analysed factors associated with BC positivity in this subgroup (online supplemental table 6). Results were similar to the main analysis, with the added importance of previous comorbidities (AOR 1.67, 95% CI 1.04 to 2.68), driven mainly by malnutrition (AOR 2.50, 95% CI 1.08 to 5.78).

## Discussion

In this multicentre surveillance study, we found that only 4.5% of BC samples for hospitalised children under 18 years old were positive for pathogens, with a large variation between countries (2–13%). *S. aureus* was the most common BC pathogen in all age groups, though less prevalent in Asian countries where Gram-negative bacteria predominated. The main factors associated with a positive BC were age (infants and 5–18 year-olds), presence of clinical severity signs, HAI and having a suspected source other than respiratory or CNS infection. Among those with a suspected respiratory infection, associated comorbidities, especially malnutrition, also increased the rates of BC positivity.

### Comparison with previous studies

The observed large variability in BC positivity rate (2–13%) found in our study has been reflected previously in children. However, our overall BC yield (4.5%) is still lower than the 19% pooled BC positivity rate (range 4–54%) reported in a LMIC 2011 meta-analysis including six Asian and six African paediatric studies.^11^ The meta-analysis included studies from the pre-pneumococcal conjugate and *H. influenzae* vaccines’ introduction, including children hospitalised for any reason (even without fever) and from both rural and tertiary urban hospitals^11^ which may explain the observed difference. Also, two thirds of the paediatric infection episodes in ACORN2 had a linked BC, a higher proportion than the under 50% reported in other studies from various settings^4 15^, which may have reduced BC yield in our study. However, as previously reported, BC positivity by site did not correlate with the proportion of infection episodes with a linked BC in ACORN2.^11^ The way BCs are performed (timing, volume and sterility) is not always optimal and this may affect BC yield and contamination rates.^51^ High contamination rates could lead to missing pathogens; however, the 9% contamination rate in our study was similar to other reports from LMICs (range 2–13%).^2 6 38 52 53^ Continuous adequate training to those performing BC may improve its yield, especially in settings with high staff turnover. All in all, the low BC yield and large variability highlight the opportunity to optimise BC sampling.

HAI was the strongest independent factor associated with BC positivity. Most previous studies did not analyse predictors for BC positivity among both community and HAI in the same population, hampering a comparison with our study. Risk factors for HAIs have been well described in children, including prolonged hospitalisation and PICU stay, comorbidities, invasive ventilation, central catheters, parenteral nutrition, blood transfusions and carbapenem use.^54^,^58^ There is a critical lack of data on paediatric HAI risk factors from LMICs. In our study, the presence of medical devices, mainly CVC and urinary catheters, was more frequently reported in children with HAI and a positive BC (online supplemental table 2). Our findings highlight the relevant role of BCs for identifying the causative bacteria during HAIs in children. In settings where BCs are billed to patients and are not performed for patients who cannot pay, these data suggest that other billing models should be explored, in particular considering BC for in-patients as an overhead since the institution’s infection prevention and control programme benefits considerably from performing BCs for patients that may have HAIs.

Clinical severity signs were also associated with BC yield. Temperature, heart rate, mental status and peripheral perfusion may be combined into a clinical severity score, showing a steady increase of the odds for a positive BC with each added clinical sign, with no relevant difference in the size of the effect of each individual sign. Clinical signs have been previously reported as predictors of BC positivity in children, both in high and LMICs.^56 59^,^66^ Abnormal temperature^6 60 66^ and altered mental status^5 6 59 61^ are the signs more frequently associated. The Yale Observation Scale (YOS) is a clinical score containing six observational items (quality of cry, reaction to parent, state variation, colour, hydration and response to social overtures), developed to identify febrile under 2-year-olds with bacterial infections.^24 67^ The YOS has been studied as a predictor for BC positivity, mainly in 3–36 month-olds with fever without a source, with varying results depending on cut-off used and study population characteristics,^63 64 68 69^ but with poor discrimination ability for invasive bacterial infections in infants <2 months old.^25^ In contrast, in our neonatal subgroup analysis, the additional three WHO general danger signs increased the probability of a positive BC, both when used individually and as a composite clinical severity score together with the previous four clinical signs, confirming the robustness of the association between the chosen clinical signs and BC positivity. Altogether, we showed that simple clinical signs that require no equipment other than a watch may be used in children of different ages to identify those with a higher BC yield.

Neonates, 1–12 month-olds and 5–18 year-olds had a higher BC positivity proportion than 1–4 year olds in our study, though only 1–12 months old and 5–18 year olds showed an association when adjusting for other factors. Age is rarely reported as being associated with BC yield, as most previous studies were targeted at specific age groups.^3 5 6 63^ Even though high BC positivity has been reported in some neonatal and infant studies,^38 39^ it may still be limited by the reduced sensitivity associated with a smaller blood sample (1–2 mL of blood),^70^ despite using appropriate BC bottle sizes. The higher positivity rate in 5–18 year-olds may be explained by the larger proportion of typhoid fever in this age group (26%), as older age has been previously identified as a predictor for BC positivity in children with typhoid fever.^27^

BC utility for respiratory infections has been questioned^33^,^36^ despite some international guidelines’ recommending them for children admitted for moderate or severe community-acquired pneumonia (CAP).^12^ In our study, suspected respiratory and CNS infections had the lowest BC yield (2.5% and 2.8%, respectively) compared with other suspected infections, a difference that remained in the final adjusted model. This low yield for CAP (1–3%) has been repeatedly reported,^15 16 71^ but BCs are still obtained in 30–50% of cases.^15 16 71^ Among children with a suspected respiratory infection, we found that those with clinical severity signs and other comorbidities, especially malnutrition, have a higher BC yield. Malnutrition has been associated with BC positivity in children irrespective of the suspected infection.^3 6 61 72 73^ Our study supports previous authors suggesting that BCs are not indicated in healthy children admitted for non-critical CAP.^16 36^ Non-culture-based diagnostics may prove useful in this population, though with lack of evidence for reducing antibiotic use in children,^74 75^ and limited availability in LMICs.

### Strengths and limitations

ACORN2 is a multicentre study that included a large number of children from nine LMICs with a high burden of infection. It was embedded into routine clinical care, resulting in real-life clinical setting data, where BC specimens were requested according to the treating physician’s criteria. We therefore feel our findings are relevant to other tertiary hospitals with BC access in LMICs in the African and Asian regions. To ensure completeness and reduce bias, information on sociodemographic and clinical factors was recorded prospectively via standardised case record forms. In addition, we performed a mixed-effects model with study site as the random effect to adjust for potential differences between the sites that could act as uncounted confounders. Sites included in ACORN2 underwent laboratory assessments and continuous laboratory quality monitoring, diagnostic stewardship and site-specific surveillance trainings. This ensured a minimum diagnostic quality across sites. This study also presents some limitations. First, we lacked information on birth parameters such as gestational age and birth weight for neonates, factors that have been associated with BC positivity.^76^ Second, the observed differences in BC yield between sites may be due to differences in clinical criteria for suspected diagnosis and in microbiological sampling, introducing selection bias despite diagnostic stewardship. Third, on occasions, there was more than one infection episode per child, but this was not accounted for in the analysis as it was not reliably collected. Fourth, while CAIs were enrolled daily, HAIs were enrolled once a week; therefore, suspected HAIs with negative BCs where antibiotics had already been stopped on the day of recruitment were not enrolled in the study, which may have biased BC positivity for HAIs towards a higher proportion. Finally, baseline information on exposures, including previous antibiotics exposure, was patient (or parent) reported, a potential source of information bias.

### Implications for practice and future research

Increasing cost-effectiveness of diagnostic methods is crucial, especially in resource-limited settings. The low BC yield reported in this multi-centre study should encourage public health entities and health centres to develop protocols to optimise BC indications. The findings from our study may be used to identify children hospitalised with a suspected infection, for which diagnostic stewardship efforts to increase BC uptake should be prioritised, and others in which it could be limited in times of financial or logistic constraints. Not performing BCs in children with suspected infections requiring hospitalisation may lead to missing bacteraemias caused by pathogens not covered by empiric antibiotic treatment. However, among those with the lowest risk, only 1% of BCs were positive. Another consideration is the impact of avoiding BCs for non-critical respiratory infections on invasive pneumococcal disease surveillance, and strategies to maintain adequate surveillance would be required. Finally, researchers may wish to take our work one step further by developing a prediction score for BC positivity to be used directly by healthcare workers.

Future research should also focus on improving culture-based, and on developing culture-independent, diagnostic tools for BSI. BC costs are partly due to expensive and not easily available consumables, such as BC bottles.^21^ Efforts should focus on developing cheaper alternatives for BC consumables. Existing blood collection diversion devices may reduce BC contamination rates,^77^ but new technologies are needed to overcome other limitations, mainly low sensitivity and slow turnaround time (24–72 hours).^51 78 79^ In addition, limiting access to over-the-counter antibiotics would reduce the proportion of children who had already received antibiotics at admission to healthcare (26% in the current study), increasing BC positivity yield. Culture-independent diagnostics that are fast, accurate, require low volumes and skills and are accessible for both high and LMICs are urgently needed,^78 79^ and healthcare settings should be actively preparing for these new technologies through education and digitalisation.^79^ These culture-independent diagnostics may be especially relevant for cases where BC yield is the lowest, such as respiratory infections. Finally, our findings using the international ACORN2 network data stress the need for microbiological surveillance collaboration networks which provide real-life international microbiological data.

### Conclusion

BC yield in children hospitalised for suspected infection is low. We identified specific groups where efforts to enhance BC testing should be prioritised, and others where it could be restricted during times of financial or logistical limitations. We highlight the relevant role of BC testing as part of the septic screening for HAI in children.

## Supplementary material

10.1136/bmjgh-2025-020448 online supplemental file 1

## Data Availability

Data are available in a public, open access repository.
