# Supplementary material for: Factors associated with positive blood cultures in children in nine African and Asian countries: the ACORN2 surveillance network
Source: BMJ Glob Health. 2025 Oct 20;10(10):e020448. doi: 10.1136/bmjgh-2025-020448 (PMC12542579; doi:10.1136/bmjgh-2025-020448)
Supplement: online supplemental file 1 [file bmjgh-10-10-s001.pdf]

Factors associated with positive BCs in children in nine African and Asian countries: the ACORN2 surveillance network

Supplementary Methods

The study's diagnostic stewardship manual (https://doi.org/10.6084/m9.figshare.27900042.v2) included the following definition for (clinical) sepsis and severe febrile illness:

Sepsis is a clinical syndrome defined as life-threatening organ dysfunction caused by a dysregulated host response to infection and is a medical emergency. There is no gold standard diagnostic test.
The original international sepsis-1 definitions included the inadequately specific systemic inflammatory response syndrome (SIRS). A score of two or more was indicative of sepsis:

- Temperature > 38°C or < 36°C
- Heart rate > 90/min
- Respiratory rate > 20/ min or $PaCO_2$ < 32 mm Hg (4.3 kPa)
- White blood cell count > 12,000 /mm^3 or < 4,000 /mm^3 or >10 % immature bands

Adult patients with suspected infection can be rapidly identified as being more likely to have poor outcomes typical of sepsis if they have at least two of the following clinical criteria that together constitute a bedside clinical score termed quick SOFA (qSOFA), which has been incorporated into the current international sepsis-3 definition.
The elements of qSOFA are:

- Respiratory rate of 22/min or greater
- Altered mentation (Glasgow Coma Score [GCS] < 15)
- Systolic blood pressure of 100 mmHg or less

The diagnosis of sepsis remains challenging in children. The UK Sepsis Trust "red flags" can be used to identify children with infection who are at risk of sepsis:

- Abnormal core temperature
    - < 36.0°C / > 38.5°C tympanic OR < 35.5°C / > 38.0°C axillary
- Inappropriate tachycardia
    - < 1y: ≥ 160 /min
    - 1-2y: ≥ 150 /min
    - 3-4y: ≥ 140 /min
    - 5y and above: ≥ 130 /min
- Altered mental state
    - GCS < 15 OR sleepiness, irritability, lethargy, floppiness
- Reduced peripheral perfusion or prolonged capillary refill time
    - Cold feet or hands OR capillary refill time ≥ 3 seconds

Also, for children aged < 5 years, "severe febrile illness" definitions* can also be used to identify children with infection who are at risk of sepsis:

- History of fever (or documented fever [≥ 38.0°C] / hypothermia [< 36.0°C]) **plus** any general danger sign:
    - Unable to drink or breastfeed
    - Vomiting everything
    - Convulsions
    - Lethargic or unconscious
    - Severe malnutrition

(*This definition is a composite of the WHO IB-VPD, IMCI 2014, and the LSTHM Fleming Fund WHO GLASS Road Map)

Sepsis in neonates (infants aged < 28 days old) may be suspected in those presenting with ≥ 1 of:

- Core temperature of > 37.5°C or < 35.5°C
- Respiratory rate of > 60/min
- Respiratory distress (severe chest in-drawing)
- Reduced movement
- Convulsion
- Poor feeding

Sepsis is strongly associated with bacteraemia. All patients suspected to have sepsis should have at least one blood culture taken. Additional investigations should be requested depending on the clinical presentation and availability e.g. chest x-ray (CXR) or other imaging, urine, sputum or wound/pus cultures, serum lactate, complete blood count (CBC), biochemistry, c-reactive protein (CRP), procalcitonin (PCT), echocardiography (if endocarditis suspected). In certain geographic locations testing for additional pathogens (e.g. malaria, dengue) may be recommended.

Supplementary Figures and Tables:

Supplementary Figure 1. ACORN surveillance workflow …………………………………………………………………... 4

Supplementary Table 1: Classification of identified bacteria from included blood cultures ……………... 5

Supplementary Table 2: Specific characteristics of included children per infection episode for hospital-acquired infections ……………………………………………………………………………………………………..………… 7

Supplementary Table 3: Pathogens identified in blood cultures of children with suspected community acquired respiratory infection, stratitied by age and for clinical severity score of 0 or ≤ 1. …………….. 7

Supplementary Table 4: Sensitivity analysis: Uncertain significance bacteria included as positive blood cultures. Mixed effects logistic regression analysis for factors associated with blood culture positivity for all children participating in ACORN study (Study site as random effect) ………………………….………. 8

Supplementary Table 5: Subgroup analysis: Neonates. Mixed effects logistic regression analysis for factors associated with blood culture positivity for all neonates participating in ACORN study (Study site as random effect) ………. 9

Supplementary Table 6: Subgroup analysis: Respiratory infections. Mixed effects logistic regression analysis for factors associated with blood culture positivity for all children with suspected respiratory infection as initial diagnosis participating in ACORN study (Study site as random effect). …………….. 10

Supplementary Figure 1. ACORN surveillance workflow

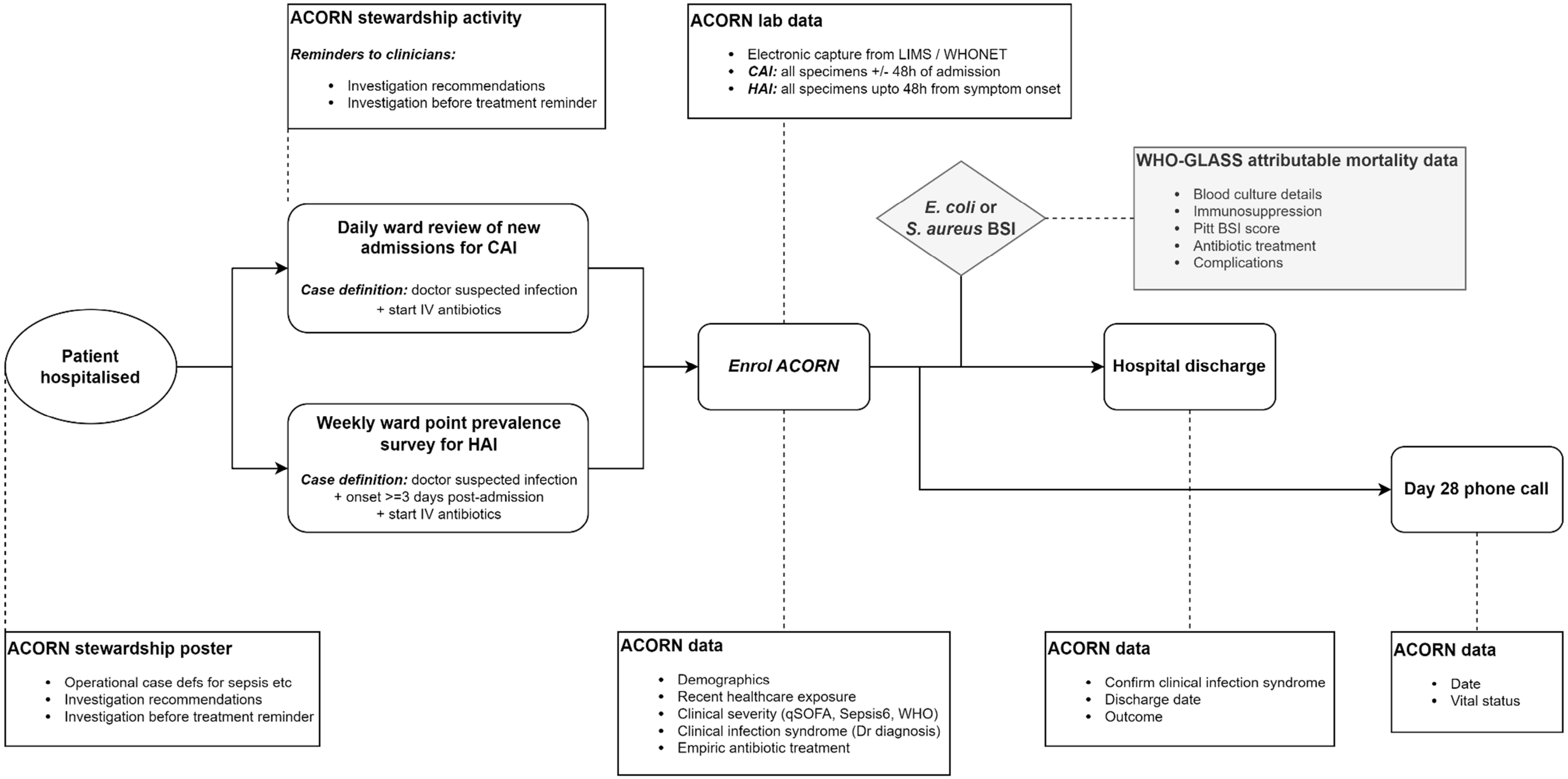

BSI: Bloodstream Infection
CAI: Community Acquired Infection
HAI: Hospital Acquired Infection
IV: Intravenous
LIMS: Laboratory Information Management System
qSOFA: quick Sequential Organ Failure Assessment
WHO GLASS: World Health Organization Global Antimicrobial Resistance and Use Surveillance System

Supplementary Table 1: Classification of identified bacteria from included blood cultures (N= 15,487)

	Classification			
**Organism name**	**Pathogen** N = 705	**Uncertain** N = 147	**Contaminant** N = 1,479	**Negative** N = 13,156
α-haemolytic strep other than pneumo	0	0	13	0
Achromobacter sp	0	2	0	0
Acinetobacter baumannii	23	0	0	0
Acinetobacter sp	0	25	0	0
Aerococcus sp	0	1	0	0
Aeromonas sp	0	3	0	0
Alloiococcus sp	0	1	0	0
Arthrobacter sp	0	0	1	0
Bacillus sp	0	0	136	0
Bordetella sp	0	1	0	0
Brevibacterium sp	0	0	4	0
Brevundimonas sp	0	1	0	0
Burkholderia cepacia	0	2	0	0
Burkholderia pseudomallei	7	0	0	0
Campylobacter jejuni	1	0	0	0
*Candida/*Yeast	17	0	0	0
Chromobacterium sp	1	0	0	0
Chryseobacterium sp	0	2	0	0
Chryseomonas sp	0	2	0	0
Citrobacter sp	8	0	0	0
Clostridium sp	0	1	0	0
Contaminant	0	0	93	0
Corynebacterium sp	0	0	3	0
Cronobacter sp	1	0	0	0
Cryptococcus sp	2	0	0	0
Cutibacterium sp	0	0	1	0
Deinococcus sp	0	1	0	0
Delftia sp	0	1	0	0
Dermacoccus sp	0	0	5	0
Diphtheroids	0	0	56	0
Elizabethkingia sp	0	2	0	0
Empedobacter sp	0	1	0	0
Enterobacter sp	20	0	0	0
Enterococcus faecalis	14	0	0	0
Enterococcus faecium	9	0	0	0
Enterococcus sp	8	0	0	0
Escherichia coli	81	0	0	0
Escherichia sp	1	0	0	0
Flavimonas sp	0	4	0	0
Gardnerella sp	0	1	0	0
GNB/GNC/GPB/GPC	0	0	80	0
Granulicatella sp	0	2	0	0
Group A Streptococcus	18	0	0	0
Group B Streptococcus	7	0	0	0
Group F Streptococcus	2	0	0	0
Group G Streptococcus	1	0	0	0
Haemophilus influenzae	5	0	0	0
Klebsiella pneumoniae	54	0	0	0

Organism name	Pathogen N = 705	Uncertain N = 147	Contaminant N = 1,479	Negative N = 13,156
Klebsiella sp	8	0	0	0
Kluyvera sp	1	0	0	0
Kocuria sp	0	0	24	0
Kytococcus schroeteri	0	0	1	0
Micrococcus sp	0	0	160	0
Mixed growth	0	0	7	0
Moraxella catarrhalis	0	2	0	0
Moraxella sp	0	2	0	0
Morganella sp	2	0	0	0
Mycobacterium sp	1	0	0	0
Myroides sp	0	1	0	0
No growth	0	0	0	13,156
Ochrobactrum sp	0	2	0	0
Other	0	4	0	0
Pandoraea sp	0	1	0	0
Pantoea sp	4	0	0	0
Pasteurella sp	0	3	0	0
Photobacterium sp	0	1	0	0
Plesiomonas shigelloides	1	0	0	0
Proteus sp	1	0	0	0
Providencia sp	2	0	0	0
Pseudomonas aeruginosa	18	0	0	0
Pseudomonas sp	0	20	0	0
Pseudoxanthomonas kaohsiungensis	0	1	0	0
Raoultella sp	2	0	0	0
Rhodococcus sp	0	2	0	0
Roseomonas sp	0	3	0	0
Rothia sp	0	1	0	0
Salmonella Paratyphi A	3	0	0	0
Salmonella sp	47	0	0	0
Salmonella Typhi	74	0	0	0
Serratia sp	2	0	0	0
Shigella sonnei	1	0	0	0
Sphingomonas sp	0	16	0	0
Staphylococcus aureus	229	0	0	0
Staphylococcus sp	0	0	895	0
Stenotrophomonas maltophilia	0	4	0	0
Streptococcus pneumoniae	28	0	0	0
Streptococcus sp	0	31	0	0
Vibrio cholerae	1	0	0	0

Note: Some children had more than one blood culture per infection episode or more than one identified bacteria species per episode. Here we present all the identified bacteria.

Supplementary Table 2: Specific characteristics of included children per infection episode for hospital-acquired infections

Characteristic	All (N = 435)	Blood culture results: Negative (N = 358)	Blood culture results: Positive (N = 77, 18%)	P value
Any medical device, n (%)	323 (74)	259 (72)	64 (83)	0.050
Peripheral VC, n (%)	301 (69)	245 (68)	56 (73)	0.5
Central VC, n (%)	23 (5.3)	15 (4.2)	8 (10)	0.044
Urinary catheter, n (%)	32 (7.4)	22 (6.1)	10 (13)	0.037
Intubation, n (%)	24 (5.5)	19 (5.3)	5 (6.5)	0.6
ICU >48h, n (%)	83 (19)	68 (19)	15 (19)	>0.9
Surgery during admission, n (%)	34 (7.8)	27 (7.5)	7 (9.1)	0.6

VC: venous catheter; ICU: intensive care unit.

Supplementary Table 3: Pathogens identified in blood cultures of children with suspected community acquired respiratory infection, stratitied by age and for clinical severity score of 0 or ≤ 1.

	Clinical severity score								
	**Any**	**0**				**≤ 1**			
**Organism name**	**Total**	**< 1 m**	**1-12 m**	**1-4 years**	**5-18 years**	**< 1 m**	**1-12 m**	**1-4 years**	**5-18 years**
**N**	5295	420	577	743	293	254	622	818	266
Acinetobacter baumannii	5	-	-	-	-	2	2	-	-
Burkholderia pseudomallei	6	-	1	1	1	-	1	2	1
Candida sp / Yeast	5	-	1	1	-	-	1	2	-
Chromobacterium sp	1	-	-	-	-	-	-	-	-
Citrobacter sp	1	-	-	-	-	-	1	-	-
Enterobacter sp	2	-	-	-	-	-	-	-	1
Enterococcus faecalis	3	1	-	-	1	2	-	-	-
Enterococcus faecium	4	1	-	1	-	2	1	1	-
Enterococcus sp	2	-	-	-	-	-	1	-	-
Escherichia coli	17	3	-	-	-	4	4	1	-
Group A Streptococcus	3	-	-	1	-	-	-	1	-
Group B Streptococcus	3	2	-	-	-	3	-	-	-
Group G Streptococcus	1	1	-	-	-	1	-	-	-
Haemophilus influenzae	1	-	1	-	-	-	1	-	-
Klebsiella pneumoniae	2	-	-	-	-	-	-	-	-
Klebsiella sp	2	-	-	-	-	-	-	-	1
Morganella sp	1	-	-	-	-	-	-	-	-
Mycobacterium sp	1	-	-	-	-	-	-	1	-
Pseudomonas aeruginosa	3	-	-	-	-	-	2	-	-
Raoultella sp	1	-	1	-	-	-	1	-	-
Salmonella sp	7	-	-	1	1	-	1	2	1
Salmonella Typhi	5	-	-	1	-	-	1	1	3
Salmonella Paratyphi A	1	-	-	-	1	-	-	-	1
Serratia sp	1	-	-	-	-	-	1	-	-
Staphylococcus aureus	34	3	3	2	1	5	8	5	2
Streptococcus pneumoniae	15	-	1	1	1	1	3	2	2
**TOTAL**	**127**	**11**	**8**	**9**	**6**	**20**	**29**	**18**	**12**

Supplementary Table 4: Sensitivity analysis: Uncertain significance bacteria included as positive blood cultures. Mixed effects logistic regression analysis for factors associated with blood culture positivity for all children participating in ACORN study (Study site and patient as random effect)

	**Blood culture results**		**Univariate**			**Multivariable**		
**Characteristic**	**Negative N = 14,552**	**Positive N = 832**	**OR**	**95% CI**	**p-value**	**AOR**	**95% CI**	**p-value**
**Sociodemographics:**	3,226 (94)	199 (5.8)						
Age groups								
<29 days	3,120 (94)	198 (6.0)	1.01	0.82-1.25	0.896	0.97	0.76-1.23	0.812
29 days-12 months	3,226 (94)	199 (5.8)	**1.39**	**1.14-1.69**	**0.001**	**1.33**	**1.06-1.66**	**0.014**
1-4 years	4,958 (96)	215 (4.2)	—	—		—	—	
5-18 years	3,248 (94)	220 (6.3)	**1.51**	**1.24-1.83**	**<0.001**	**1.62**	**1.30-2.01**	**<0.001**
Female sex	6,298 (95)	358 (5.4)	0.95	0.83-1.10	0.513			
**Recent HC exposure**	2,891 (95)	155 (5.1)	0.99	0.80-1.23	0.931			
**Comorbidities**								
Any	2,570 (94)	174 (6.3)	1.14	0.94-1.39	0.175			
Cancer	393 (93)	28 (6.7)	1.24	0.81-1.89	0.324			
Malnutrition	406 (92)	36 (8.1)	1.35	0.93-1.98	0.116			
**Infection episode:**								
Hospital acquired infection	352 (81)	83 (19)	**2.88**	**2.20-3.78**	**<0.001**	**3.05**	**2.30-4.06**	**<0.001**
Suspected diagnosis								
Respiratory infection	5,212 (97)	184 (3.4)	—	—		—	—	
Sepsis	4,177 (92)	339 (7.5)	**1.72**	**1.41-2.10**	**<0.001**	**2.09**	**1.67-2.61**	**<0.001**
GI or abdominal infection	1,206 (92)	102 (7.8)	**2.21**	**1.72-2.85**	**<0.001**	**2.36**	**1.78-3.13**	**<0.001**
CNS infection	945 (97)	34 (3.5)	0.79	0.54-1.15	0.212	0.74	0.49-1.13	0.166
SST or bone infection	531 (91)	54 (9.2)	2.71	**1.96-3.75**	**<0.001**	**3.64**	**2.57-5.14**	**<0.001**
Genitourinary infection	340 (93)	25 (6.8)	2.03	**1.30-3.15**	**0.002**	**2.22**	**1.39-3.56**	**<0.001**
Other or unknown	2,141 (96)	94 (4.2)	1.34	1.03-1.76	0.031	1.26	0.93-1.72	0.136
Clinical characteristics:								
Abnormal temperature	5,068 (93)	353 (6.5)	**1.52**	**1.31-1.77**	**<0.001**			
Tachycardia	3,236 (94)	220 (6.4)	**1.24**	**1.05-1.47**	**0.010**			
Altered mental status	3,238 (94)	206 (6.0)	**1.23**	**1.01-1.49**	**0.035**			
Reduced peripheral perfusion	1,637 (94)	100 (5.8)	**1.29**	**1.01-1.64**	**0.039**			
Clinical severity score	1 (0-2)	1 (0-2)	**1.23**	**1.14-1.33**	**<0.001**	**1.29**	**1.18-1.40**	**<0.001**
BC within 24h of admission	13,880 (95)	797 (5.4)	1.23	0.85-1.78	0.267			
Antibiotic 24h before BC	3,360 (95)	167 (4.7)	1.11	0.87-1.42	0.394			

BC = blood culture; CI = Confidence Interval; CNS = central nervous system; HC = health care; GI= gastrointestinal; OR = Odds Ratio; SST = skin and soft tissue infection

Supplementary Table 5: Subgroup analysis: Neonates. Mixed effects logistic regression analysis for factors associated with blood culture positivity for all neonates participating in ACORN study (Study site and patient as random effect)

	Blood culture results		Univariate			Multivariable		
**Characteristic**	**Negative N = 3141**	**Positive N = 177**	**OR**	**95% CI**	**p-value**	**OR**	**95% CI**	**p-value**
**Sociodemographics:**								
Age (days)	3 (1-10)	4 (2-11)	1.02	1.00-1.04	0.049	1.00	0.98-1.03	0.710
Female sex	1427 (95)	76 (5.1)	0.88	0.65-1.21	0.439			
**Recent HC exposure**	450 (97)	14 (3.0)	1.19	0.57-2.47	0.646			
**Comorbidity (any)**	22 (92)	2 (8.3)	1.06	0.24-4.69	0.937			
**Infection episode:**								
Hospital acquired infection	140 (77)	41 (23)	**5.02**	**2.93-8.59**	**<0.001**	**4.46**	**2.56-7.77**	**<0.001**
Suspected diagnosis								
Respiratory infection	747 (97)	24 (3.1)	—	—		—	—	
Sepsis	1952 (94)	128 (6.2)	**1.79**	**1.09-2.96**	**0.022**	**1.68**	**1.01-2.81**	**0.048**
SST or bone infection	125 (95)	7 (5.3)	**2.62**	**1.05-6.50**	**0.038**	**2.64**	**1.01-6.88**	**0.047**
GI or abdominal infection	119 (94)	7 (5.6)	**3.53**	**1.37-9.14**	**0.009**	2.65	0.97-7.25	0.057
CNS infection	42 (89)	5 (11)	2.76	0.95-8.02	0.063	2.48	0.81-7.62	0.112
Other or unknown	143 (96)	6 (4.0)	1.12	0.44-2.82	0.815	0.91	0.34-2.43	0.854
Clinical characteristics:								
Abnormal temperature	724 (93)	55 (7.1)	**1.62**	**1.14-2.31**	**0.007**			
Tachycardia	475 (92)	44 (8.5)	1.40	0.96-2.04	0.081			
Altered mental status	450 (91)	45 (9.1)	1.35	0.91-2.00	0.136			
Reduced peripheral perfusion	185 (91)	19 (9.3)	1.02	0.60-1.75	0.935			
Reduced activity	536 (92)	49 (8.4)	1.15	0.80-1.66	0.456			
Feeding difficulty	833 (91)	78 (8.6)	**1.74**	**1.24-2.45**	**0.001**			
Convulsions	96 (87)	14 (13)	**2.18**	**1.19-3.97**	**0.011**			
Clinical severity score	0 (0-1)	1 (0-1)	**1.25**	**1.06-1.49**	**0.009**			
Clinical neonates score	1 (0-2)	1 (0-3)	**1.20**	**1.08-1.33**	**<0.001**	**1.18**	**1.05-1.32**	**0.005**
BC within 24h of admission	2932 (94)	171 (5.5)	1.80	0.78-4.12	0.167			
Antibiotic 24h before BC	461 (92)	42 (8.3)	**2.14**	**1.34-3.43**	**0.001**			

BC = blood culture; CI = Confidence Interval; CNS = central nervous system; HC = health care; GI= gastrointestinal; OR = Odds Ratio; SST = skin and soft tissue infection

Supplementary Table 6: Subgroup analysis: Respiratory infections. Mixed effects logistic regression analysis for factors associated with blood culture positivity for all children with suspected respiratory infection as initial diagnosis participating in ACORN study (Study site as random effect).

A. Multivariable 1: Including 'Any comorbidity' as covariate

	**Blood culture results**		**Univariate**			**Multivariable 1**		
**Characteristic**	**Negative N = 5260**	**Positive N = 136**	**OR**	**95% CI**	**p-value**	**OR**	**95% CI**	**p-value**
**Demographics:**								
Age groups								
<29 days	747 (97)	24 (3.1)	1.29	0.69-2.42	0.431	1.54	0.79-3.00	0.208
29 days-12 months	1,643 (97)	51 (3.0)	**1.83**	**1.18-2.84**	**0.007**	**1.90**	**1.21-2.96**	**0.005**
1-4 years	2,115 (98)	34 (1.6)	—	—		—	—	
5-18 years	755 (97)	27 (3.5)	**2.14**	**1.29-3.57**	**0.003**	**1.96**	**1.16-3.30**	**0.012**
Female sex	2,256 (97)	61 (2.6)	1.06	0.76-1.49	0.729			
**Recent HC exposure**	1,170 (98)	27 (2.3)	1.06	0.66-1.70	0.804			
**Comorbidities**								
Any	638 (96)	28 (4.2)	**1.84**	**1.17-2.87**	**0.008**	**1.67**	**1.04-2.68**	**0.034**
Cancer	80 (99)	1 (1.2)	0.57	0.08-4.17	0.583			
Malnutrition	110 (94)	7 (6.0)	**2.56**	**1.12-5.83**	**0.026**			
**Infection episode:**								
Hospital acquired infection	92 (91)	9 (8.9)	**3.68**	**1.79-7.55**	**<0.001**	**3.11**	**1.50-6.45**	**0.002**
Clinical characteristics:								
Abnormal temperature	1,867 (97)	64 (3.3)	**1.48**	**1.04-2.10**	**0.030**			
Tachycardia	1,540 (97)	45 (2.8)	1.40	0.96-2.05	0.077			
Altered mental status	1,110 (96)	46 (4.0)	**1.97**	**1.26-3.09**	**0.003**			
Reduced peripheral perfusion	288 (97)	10 (3.4)	1.49	0.76-2.92	0.251			
Clinical severity score	1 (0-1)	1 (0-2)	**1.41**	**1.17-1.70**	**<0.001**	**1.35**	**1.12-1.63**	**0.002**
BC within 24h of admission	4,989 (97)	128 (2.5)	0.92	0.44-1.92	0.817			
Antibiotic 24h before BC	1,050 (98)	22 (2.1)	1.09	0.61-1.92	0.780			

BC = blood culture; CI = Confidence Interval; HC = health care; OR = Odds Ratio

B. Multivariable2: including 'Malnutrition' (instead of 'Any comorbidity') as covariate

	**Blood culture results**		**Univariate**			**Multivariable 2**		
**Characteristic**	**Negative N = 5260**	**Positive N = 136**	**OR**	**95% CI**	**p-value**	**OR**	**95% CI**	**p-value**
**Demographics:**								
Age groups								
<29 days	747 (97)	24 (3.1)	1.29	0.69-2.42	0.431	1.43	0.74-2.77	0.287
29 days-12 months	1,643 (97)	51 (3.0)	**1.83**	**1.18-2.84**	**0.007**	**1.81**	**1.17-2.83**	**0.008**
1-4 years	2,115 (98)	34 (1.6)	—	—		—	—	
5-18 years	755 (97)	27 (3.5)	**2.14**	**1.29-3.57**	**0.003**	**2.14**	**1.27-3.59**	**0.004**
Female sex	2,256 (97)	61 (2.6)	1.06	0.76-1.49	0.729			
**Recent HC exposure**	1,170 (98)	27 (2.3)	1.06	0.66-1.70	0.804			
**Comorbidities**								
Any	638 (96)	28 (4.2)	**1.84**	**1.17-2.87**	**0.008**			
Cancer	80 (99)	1 (1.2)	0.57	0.08-4.17	0.583			
Malnutrition	110 (94)	7 (6.0)	**2.56**	**1.12-5.83**	**0.026**	**2.50**	**1.08-5.78**	**0.032**
**Infection episode:**								
Hospital acquired infection	92 (91)	9 (8.9)	**3.68**	**1.79-7.55**	**<0.001**	**3.15**	**1.52-6.52**	**0.002**
Clinical characteristics:								
Abnormal temperature	1,867 (97)	64 (3.3)	**1.48**	**1.04-2.10**	**0.030**			
Tachycardia	1,540 (97)	45 (2.8)	1.40	0.96-2.05	0.077			
Altered mental status	1,110 (96)	46 (4.0)	**1.97**	**1.26-3.09**	**0.003**			
Reduced peripheral perfusion	288 (97)	10 (3.4)	1.49	0.76-2.92	0.251			
Clinical severity score	1 (0-1)	1 (0-2)	**1.41**	**1.17-1.70**	**<0.001**	**1.37**	**1.14-1.66**	**0.001**
BC within 24h of admission	4,989 (97)	128 (2.5)	0.92	0.44-1.92	0.817			
Antibiotic 24h before BC	1,050 (98)	22 (2.1)	1.09	0.61-1.92	0.780			

BC = blood culture; CI = Confidence Interval; HC = health care; OR = Odds Ratio
